# Isolated Small Bowel Mesentery Injury After Steering Wheel Trauma

**DOI:** 10.5812/traumamon.4960

**Published:** 2012-07-31

**Authors:** Imtiaz Wani, Rayees A Bhat, Shayiq Wani, Nawab Khan, Rauf A Wani, Fazal Q Parray

**Affiliations:** 1 Department of surgery SKIMS, Kashmir, India; 2 Department of surgery SMHS Hospital, Kashmir, India

**Keywords:** Intestine, Wound and Injuries, Mesentery

## Abstract

**Background:**

Isolated small gut mesentery injury after blunt abdominal trauma from the steering wheel in road traffic accidents is rare. These are always challenging to diagnose and pose a diagnostic dilemma.

**Objectives:**

To study the pattern of small gut mesenteric injury by steering wheel blunt abdominal trauma in road traffic accidents in patients who had laparotomy.

**Patients and Methods:**

A 10-year retrospective study was done to study isolated small gut mesentery injury.

**Results:**

All patients who had isolated mesenteric small gut injury were males. Jejunal mesentery was involved in 13 whereas 4 had ileal mesentery injury. Tear were longitudinal or transverse.

**Conclusions:**

Isolated small mesentery injury after blunt abdominal trauma from the steering wheel in road traffic accidents is rare. Tears are either longitudinal or transverse. Suture repair is to be done. Delay in reaching hospital or reaching the diagnosis could lead to morbidity and mortality. Isolated mesenteric injury should be considered in any patient with blunt abdominal trauma from steering wheel injury with no evidence of any solid organ injury in unstable patients.

## 1. Background

Mesenteric injury from blunt abdominal trauma is rare and can be difficult to diagnose (1). Steering wheel injuries leading to abdominal organ injury is rare. Steering wheel induced lower abdominal injuries encompasses contusions and lacerations of varying severity and frequency in the mesentery, the small and large bowels, the spleen, and the cecum (2). Approximately 13.5 % of all patients undergoing laparotomy after blunt abdominal trauma had small and large bowel mesenteric injuries (3, 4). In mesenteric injuries, small bowel injuries are more frequent than colon injuries, probably due to the factors including location and lack of redundancy, which prevents formation of closed loops (5). Isolated small bowel mesentery injury is rare. Mesenteric injury may not cause clinical manifestations (6). Isolated lesions of the small intestine can occur after steering wheel trauma and the mesenteric injuries might remain undiagnosed after blunt abdominal trauma (7). Jejunal mesentery injury may be either longitudinal or transverse. Delay in diagnosis is significantly associated with morbidity and prolonged duration of hospital stay (8). A high clinical suspicion is prime in diagnosis. Surgical intervention is necessary for management.

## 2. Objectives

To study the pattern of small gut mesenteric injury by steering wheel blunt abdominal trauma in road traffic accidents in patients who had laparotomy.

## 3. Patients and Methods

A 10-year retrospective study from 2001 to 2011 was done at the Sher-i-Kashmir Institute of Medical Sciences, a tertiary care hospital. Patients having blunt abdominal trauma from steering wheel injury in road traffic accidents were assessed to study the pattern of isolated small gut mesenteric injuries. Medical records of each patient was studied with regard to age, sex, clinical presentation, radiological method used and details of preoperative findings. All had clinical and radiological confirmation for exploratory laparotomy.

## 4. Results

Seventeen patients were studied. Age ranged from 18 - 58 years (mean 35.2 ± 10.3). All were driving vehicles. No patient was under influence of alcohol. None were females. None had any protective device (i.e. airbags) for preventing steering wheel injury. All had isolated blunt abdominal trauma from the steering wheel impact. Time lapse before reporting varied from 30 minutes to 6 hours (Mean 121 min ± 84). Number of tears varied from single to multiple. Size varied from 1 cm to complete mesenteric tear extending from root of mesentery to mesenteric border of the small gut. Jejunal mesentery was torn in 13 patients whereas 4 had tears in the ileal mesentery near the terminal ileum. Amount of blood loss varied from 400 ml to 3 liters in the peritoneal cavity (mean 1055 ± 615 cc). Longitudinal tear was seen in 11 patients, transverse tears were present in 6 patients. All were sutured. One patient had died due to a complete tear who referred after 6 hours and had 3 liters of blood loss in the abdomen.

## 5. Discussion

Blunt trauma to the abdomen from steering wheel impact leading to mesenteric injury is a grave injury. Mesenteric tears are notorious tears and these are deceleration injuries. Mesentery of small intestine is most frequently injured; and isolated injuries of the small bowel mesentery due to blunt abdominal trauma are rare (9).

Clinically isolated mesenteric injuries present as follows (10).

1) Immediate–Due to bleeding. Signs of continuous bleeding and peritoneal irritation are present, making early laparotomy imperative.

2) Delayed- Due to bowel infarction. The patient may present between 12 hours to 5 days following injury.

3) Due to bowel stenosis or adhesion formation. The time of presentation is between 5 to 8 weeks after injury.

Small gut mesenteric tears can be longitudinal or transverse. Mesenteric tears can extend from the root of mesentery to the gut margin. These can be single or multiple tears. Longitudinal tears are more common than transverse tears. Longitudinal tears can be single or multiple; the length of involvement can be a small or extensive. In transverse tears even a single vessel bleeder can be alarming. Whereas in longitudinal tears, the length of tears vary from small to full length (from gut margin to the root of the mesentery). Both tears are more distally located.

Intensity, extent of tears and length depends directly on intensity of trauma, type of steering wheel and size, physique of the driver, speed of the vehicle and contents of the gut. Type of tear depend on whether the spokes of the steering wheel had impact or the rim had impact or the both. The area of steering wheel having impact with the area of the abdomen and the angle at which the abdomen strikes the steering wheel and patient’s momentum may be influential. Steering wheel stiffness has been found to be the primary determinant of abdominal injury severity (10).

Anatomical location: In upper part of peritoneal cavity of jejunum favors steering wheel injury compared to ileum lying in lower part of abdominal cavity and in pelvis. Position of steering predominantly affects jejunum in steering wheel injury. Jejunal arcades are one or two having long branches with relatively low fat deposits in the mesentery renders it easily vulnerability to easy damage in steering wheel injury. Numerous terminal branches from multiple arcades (3-4) in the ileum may bleed rapidly and account for shock. An injury in mesenteric laden fat minute vasculature often causes diffuse oozing. Injury may involve anterior or posterior mesentery. Longitudinal tears if extensive can involve the root of mesentery and superior mesenteric vessels. Most of the time minute arcades laden in mesentery often bleed in tears and suture ligation is all that needs to be done. Multiple longitudinal tears in mesentery presenting both in jejunum and ileum require suture repair. One patient had terminal branch bleed from arcade. Delayed diagnosis of patients leads to intestinal infarction and requires bowel resection. Mesenteric vascular injury may induce chronic ischemia of the corresponding segment of small bowel, inducing secondary thickening of the bowel wall and intestinal occlusion (1, 11). Mesenteric injury after blunt abdominal trauma may lead to double ischemic ileal stenosis (12). Previous trauma secondary to steering wheel impact may lead incipient hemorrhage and may present later as adhesive intestinal obstruction. Diagnosis is on clinical suspicion in order to reduce the complications following delayed treatment of these injuries. The tears are challenging to manage and treat. Delay in reaching hospital after mesenteric injury may be paramount in determining morbidity and mortality. Profuse blood loss in extensive tears may lead to shock and death. Every attempt should be made to reach a diagnosis within 6 hours of admission to the trauma unit (13). The presence of a moderate to large volume of intraperitoneal fluid without visible solid organ injury is an important sign of bowel or mesenteric injury (14). Major changes in steering wheel columns (collapsibility) and personal protective devices (near standardization of airbags.) have lessened occurrence of mesenteric injury in present times. A timely diagnosis can be a determining survival factor. Rapidity with which repair is done may also be a determining factor in mortality and morbidity.

Isolated small mesentery injury after blunt abdominal trauma from the steering wheel in road traffic accidents is rare. Isolated small gut mesenteric injury should be considered in any patient with blunt abdominal trauma after steering wheel injury with no evidence of any solid organ injury in an unstable patient. Jejunal mesenteric tears are more common than ileal mesenteric tears from steering wheel injury. They tears can be longitudinal or transverse. Suture ligation is all that is required in these tears. Delayed reporting to hospital or delay in diagnosis of mesenteric injury and late operative intervention increases morbidity and hospital stay.
